# ATF4 promotes lung cancer cell proliferation and invasion partially through regulating Wnt/β-catenin signaling

**DOI:** 10.7150/ijms.43167

**Published:** 2021-01-29

**Authors:** Jiang Du, Haifeng Liu, Xiaoyun Mao, Yanan Qin, Chuifeng Fan

**Affiliations:** 1 Department of Pathology, First affiliated hospital and College of Basic Medical Sciences of China Medical University, 110001, Shenyang, China.; 2 Department of Breast Surgery, Department of Surgical Oncology, Research Unit of General Surgery, the First Affiliated Hospital of China Medical University, 110001, Shenyang, China.

**Keywords:** ATF4, β-catenin, lung cancer, proliferation, invasion

## Abstract

Activating transcription factor 4 (ATF4) is a member of the cAMP response element binding (CREB) protein family and has been reported to participate in cancer progression; however, its molecular mechanism is not fully understood. In this study, we investigated the function of ATF4 in non-small cell lung cancer and its molecular regulation. We detected cytoplasmic and nuclear ATF4 expression in lung cancer A549, H1299, and LK2 cells, and the total expression of ATF4 was higher than that in HBE cells (*p* < 0.05). Higher nuclear ATF4 expression was detected in all these cells compared to cytoplasmic ATF4 expression (*p* < 0.05). Overexpression of ATF4 in A549 cells significantly promoted cancer cell growth and invasion (*p* < 0.05). Expression of Wnt signaling molecules, including β-catenin, MMP7, and cyclin D1, and the activity of canonical Wnt signaling were also significantly promoted by ATF4 (*p* < 0.05). ICG001, a canonical Wnt signaling inhibitor that selectively inhibits β-catenin/ cyclic adenosine monophosphate response element binding protein (CBP) interaction, significantly inhibited cancer cell invasion and Wnt signaling. The function of ATF4 was also significantly inhibited by ICG001 (*p* < 0.05). However, compared to treatment with ICG001, the invasion ability of cancer cells treated with both ICG001 and ATF4 cDNA significantly increased (*p* < 0.05), which indicates that the function of ATF4 was not dependent only on Wnt/β-catenin signaling. The function of ATF4 in the regulation of β-catenin expression was not significantly affected by ICG001 (*p* > 0.05). The function of ATF4 to promote the activity of Wnt/β-catenin signaling in cancer cells was abolished by treatment with ICG001 (*p* > 0.05). These results indicate that ATF4 may contribute to lung cancer progression at least partly by regulating Wnt/β-catenin signaling.

## Introduction

Lung cancer is one of the most common tumors worldwide 1. Its pathological types mainly include small cell carcinoma and non-small cell lung carcinoma (NSCLC). The main pathological types of NSCLC are squamous cell carcinoma (SCC) and adenocarcinoma 2. At present, the main treatment for non-small cell lung cancer is surgery. However, molecular targeted therapy has become an important treatment for patients with advanced NSCLC, which has played a significant role in improving the survival of patients 3, 4. Understanding the molecular mechanism of lung cancer invasion and metastasis is an indispensable basis for the development of targeted therapy.

The Wnt gene was first identified in mice, called Int-1. It is named Wnt because of its high homology with the wingless gene of *Drosophila melanogaster* 5, 6. The Wnt pathway plays an important role in regulating embryonic development and cell differentiation 5, 6. Abnormalities in Wnt/β-catenin signaling have been found in many tumors, including NSCLC, and play important roles in cancer progression 7. Activating transcription factor 4 (ATF4) is a stress response protein induced by hypoxia and other factors 8. It has been found to regulate cell damage under conditions such as hypoxia and chemotherapy 8-10. ATF4 is also involved in the differentiation of cells, including osteocytes 11. The role of ATF4 in malignant tumors has been widely reported, but its role is still controversial, which may be related to specific cell types and molecular context. Zeng's study indicates that ATF4 promotes invasion and progression of breast carcinomas 12, and Chen's experiments confirmed that ATF4 promotes proliferation and invasion of gliomas 13. Others have provided evidence of its anti-cancer function 9,14, and Dai proved that ATF4 inhibits the progression of pancreatic cancer 14. ATF4 participates in the regulation of many molecular pathways. Zeng proved that ATF4 contributes to the malignant features of breast cancer by regulating E-cadherin 12. Other molecular links include PERK, MYC, and CCL2 14-16. Some experiments show that ATF4 regulates Wnt/β-catenin signaling 11, 17. However, its role and molecular mechanism in lung cancer cells are still unclear. In this study, we focused on the function of ATF4 and its role in the activation of Wnt/β-catenin signaling.

## Materials and methods

### Cell culture and transfection

We used human bronchial epithelial HBE cells and NSCLC A549, H1299, and LK2 cells. HBE, A549, and NCI-H1299 cells were obtained from the American Type Culture Collection (Manassas, VA), and LK2 cells were obtained from the Japanese Collection of Research Bioresources Cell Bank (JCRB). Cells were cultured in RPMI 1640 at 37 °C in a humidified atmosphere with 5% CO_2_. The ATF4 cDNA clone was transfected using Lipofectamine 2000 (Invitrogen, Carlsbad, CA, USA) according to the manufacturer's instructions. The medium was supplemented 12 h after transfection. G418 was used for post-transfection selection.

### Fluorescence microscopy

Fluorescence microscopy was performed as previously described 18. Cells were treated with 4% paraformaldehyde for 30 min and then washed twice with PBS. The cells were then treated with 0.1% Triton X-100 for 2 min. After washing twice with PBS, the cells were treated with 1% BSA. They were then incubated with primary antibody. The antibody for detecting ATF4 expression was purchased from Santa Cruz (sc-22800, USA, 1:500). The secondary antibodies (1:200) were conjugated to FITC. DAPI was used for nuclei counterstaining. Immunofluorescent staining of the cells was performed using an Olympus IX51 fluorescent microscope (Olympus, Tokyo, Japan). Images were captured using a CoolPIX 5400 camera (Nikon, Japan) fixed in a fluorescent microscope.

### Western blotting

Western blotting was performed as described previously 18. Protein samples were collected, concentrated, and SDS-PAGE protein loading buffer was added. Samples were analyzed using SDS-PAGE and then transferred to a PVDF membrane. The membrane was immediately placed in the prepared western washing solution and rinsed for 1-2 min. The membrane was then treated with blocking solution, followed by incubation with the first antibody overnight. The next day it was incubated with the second antibody (1:2500) at room temperature for 2 h. The primary antibodies used were: anti-ATF4 (sc-22800, Santa Cruz, USA, 1:200), anti-β-catenin (sc-393501, anti-Santa Cruz, USA, 1:200), anti-cyclin D1 (sc-20044, Santa Cruz, 1:200), anti-MMP7 (sc-515703, Santa Cruz, 1:200), anti-Histone H3(sc-517576, Santa Cruz, 1:500) and anti-GADPH (sc-59540, Santa Cruz, USA, 1:1000). The membrane was treated with enhanced chemiluminescence (ECL) for protein bands, and the signals were detected using a BioImaging System. To determine the relative protein expression in nucleus and cytoplasm separately, nuclear and cytoplasmic protein samples were prepared by using the Nuclear plasma separation Kit (Beyotime, China).

### Colony formation assay

Exponentially growing cells (1 × 10^3^) were suspended after digestion with trypsin, added to a culture dish, and gently shaken to allow the cells to disperse evenly. Cells were cultured in an incubator for 2-3 weeks, and the culture medium was changed according to the pH value. Colony formation was observed regularly during cell culture, and the culture was terminated when visible clones appeared in the culture dish. After washing and fixation with formaldehyde, the samples were stained with Giemsa for 10 min. The number of clones with more than 50 cells was counted under a microscope.

### Matrigel invasion assay

Matrigel invasion assays were performed as previously described 18. Cell invasiveness was determined using 24-well Transwell (Corning, NY) and Matrigel (BD Bioscience). Frozen matrigel was thawed at 4 °C overnight, diluted in serum-free medium (1:3), added (100 µL) to the upper chamber, and incubated at 37 °C for at least 5 h until the matrigel became solid. A cell suspension in 100 µL containing 3 × 10^5^ cells was added into the upper chamber. Medium containing 20% FBS was added to the lower chamber, and the cells incubated at 37 °C in an incubator for 24 h. The cells were then washed with PBS, fixed with 5% glutaraldehyde, and stained with 2% crystal violet. After cleaning, the cells on the upper surface were wiped off with a cotton ball, and those that appeared on the lower surface of the filter were observed under the microscope.

### Dual-luciferase assay

Dual-luciferase assays were performed as described by Zhao 19 and Prévotat 6. Plasmids with the reporter gene included pGL3-OT expressing sites for a wild-type TCF-4 binding for fluorescence detection and pGL3-OF with mutated TCF-4 binding sites. pRL-TK was used as the control plasmid. A dual-luciferase assay system (Promega, Madison, WI) was used to evaluate the expression of reporter genes. To pre-stimulate the Wnt signaling pathway, 100 ng/mL of recombinant human Wnt3a (R&D Systems) was used.

### Statistical analysis

SPSS software (v22.0, SPSS Inc., Chicago, IL, USA) was used for all data analyses. The Student's t-test was used to compare data from the densitometry analysis. All data from *in vitro* experiments were expressed as means ± standard deviation (S.D.) and repeated at least three times. P-values were considered significant when less than 0.05.

## Results

### ATF4 overexpression promotes growth and invasion of A549 lung cancer cells

**Figure 1:**
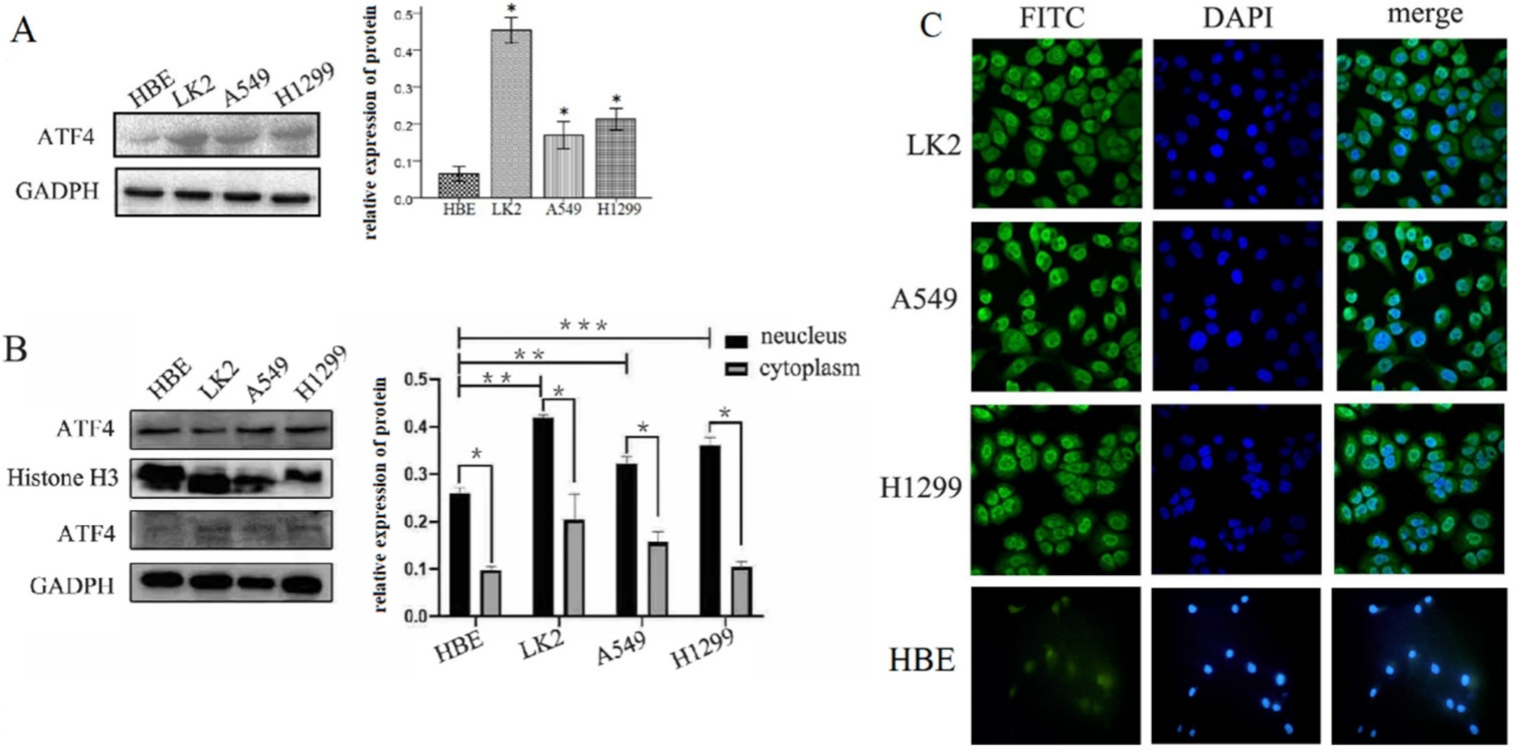
**Detection of ATF4 expression in HBE and cancer cells.** (A)Western blot study showed an overexpression of ATF4 in cancer cells including A549, H1299 and LK2 compared to normal bronchial epithelial cell HBE (*p<0.05). (B) Nuclear plasma separation experiments showed that ATF4 expression in the nucleus was significantly higher than that in the cytoplasm in cancer cells and HBE cells (*p < 0.05). ATF4 expression in A549 and LK2 cells was significantly higher than that in HBE cells, both in the nucleus and cytoplasm (**p < 0.05). ATF4 expression in nucleus in H1299 cells was significantly higher than that in HBE cells (***p < 0.05). (C) Fluorescence microscopy also shows both nuclear and cytoplasmic expression of ATF4 in HBE and cancer cells. The experiments were repeated at least 3 times.

**Figure 2:**
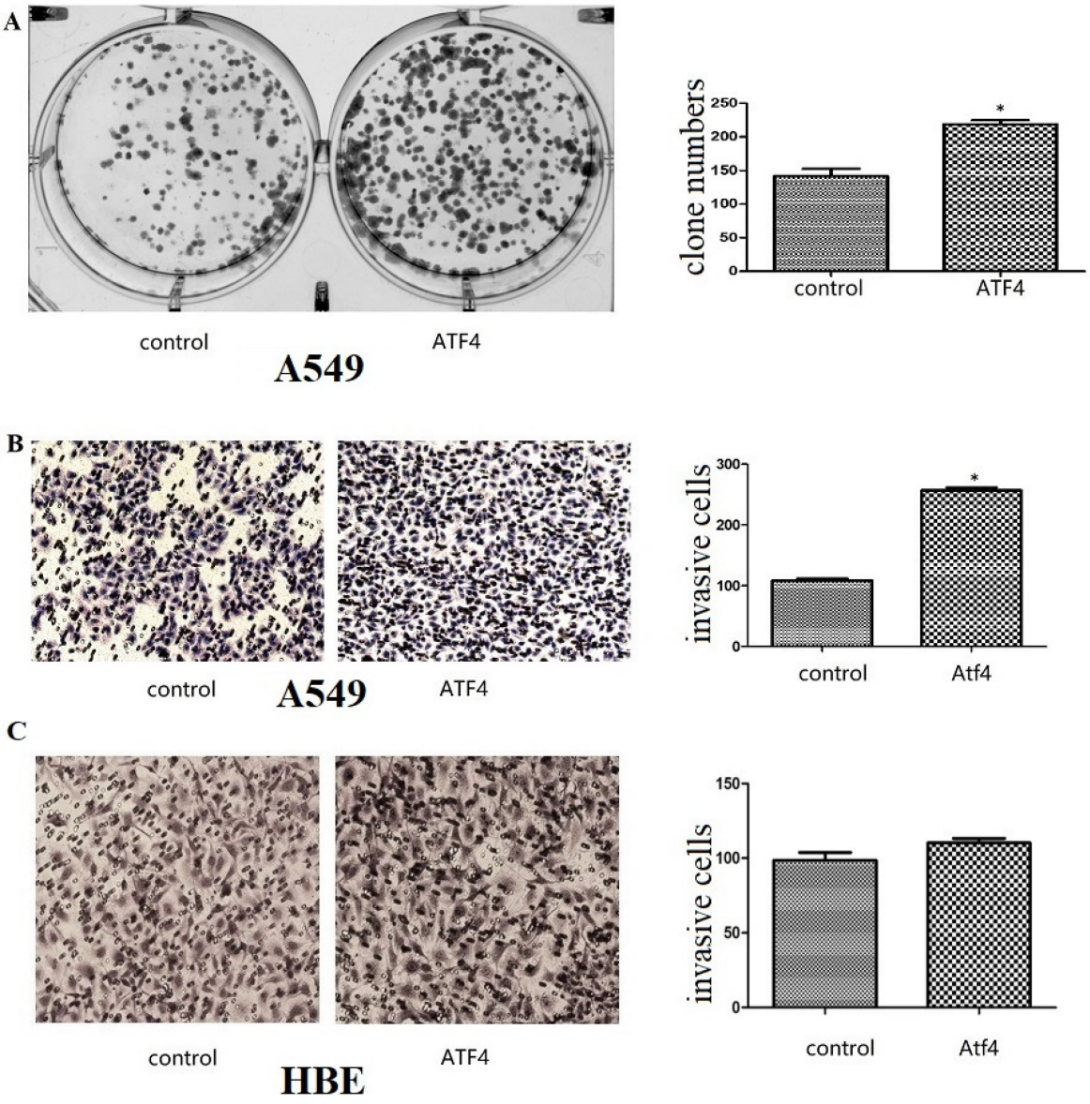
** The function of ATF4 to regulate cancer cell biology.** (A,B) Overexpression of ATF4 significantly promoted cancer cell growth (A, colony formation study) and invasion (B, Matrigel invasion assay) (*p*<0.05). (C) ATF4 overexpression in HBE cells did not significantly promote cell invasion (*p*>0.05). The experiments were repeated at least 3 times. The group of “ATF4” refers to the group with overexpression of ATF4 in A549 cells and HBE cells.

We detected ATF4 expression in human bronchial HBE cells and non-small cell lung cancer cells, including A549, H1299, and LK2 cells *in vitro* using western blotting (Figure 1A and B) and fluorescence microscopy (Figure 1C). Western blotting analysis shows that ATF4 was highly expressed in lung cancer cells compared to HBE cells (*p* < 0.05) (Figure 1A). Nucleus plasma separation experiments showed that ATF4 expression in the nucleus was significantly higher than that in the cytoplasm in all these cells (*p* < 0.05) (Figure 1B). ATF4 expression in cancer cells including A549 and LK2 was significantly higher than that in HBE cells, both in the nucleus and cytoplasm (*p* < 0.05) (Figure 1B). ATF4 expression in nucleus in H1299 cells was significantly higher than that in HBE cells (Figure 1B). To investigate the molecular mechanism underlying the function of ATF4, we selected A549 cells to overexpress ATF4. The results show that ATF4 overexpression significantly promoted cancer cell growth (Figure 2A) and invasion (Figure 2B) (*p* < 0.05). However, ATF4 overexpression in HBE cells did not significantly promote cell invasion in HBE cells (*p* > 0.05, Figure 2C), which indicates different functions of ATF4 in cancer cells compared with bronchial epithelial cells.

### ATF4 regulates β-catenin expression and Wnt/β-catenin signaling

**Figure 3:**
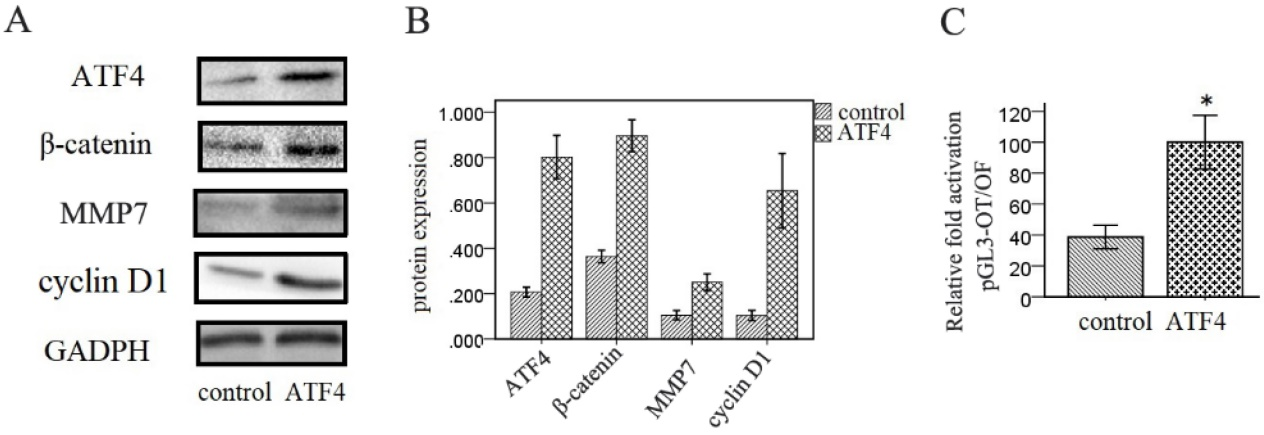
** Regulation of Wnt/β-catenin signaling by ATF4.** (A,B,) Overexpression of ATF4 significantly upregulated the expression of Wnt signaling molecules including β-catenin, MMP7 and cyclin D1. (C) Dual-luciferase assays showed that overexpression of ATF4 upregulated the activity of Wnt/β-catenin signaling (*p*<0.05). The group of “ATF4” refers to the group with overexpression of ATF4 in A549 cells. The experiments were repeated at least 3 times.

Next, we investigated the effects of ATF4 on the Wnt signaling pathway in A549 cells. Western blotting analysis shows that overexpression of ATF4 significantly upregulated the expression of β-catenin, a key molecule in the canonical Wnt pathway, and of downstream molecules including MMP7 and cyclin D1 (*p* < 0.05) (Figure 3A and 3B). Dual-luciferase assays also show that ATF4 overexpression significantly increased the activity of the canonical Wnt/β-catenin signaling pathway (*p* < 0.05) (Figure 3C).

### Wnt/β-catenin inhibitor ICG001 prevents ATF4 from promoting cancer cell invasion and activating the Wnt/β-catenin signaling pathway

**Figure 4:**
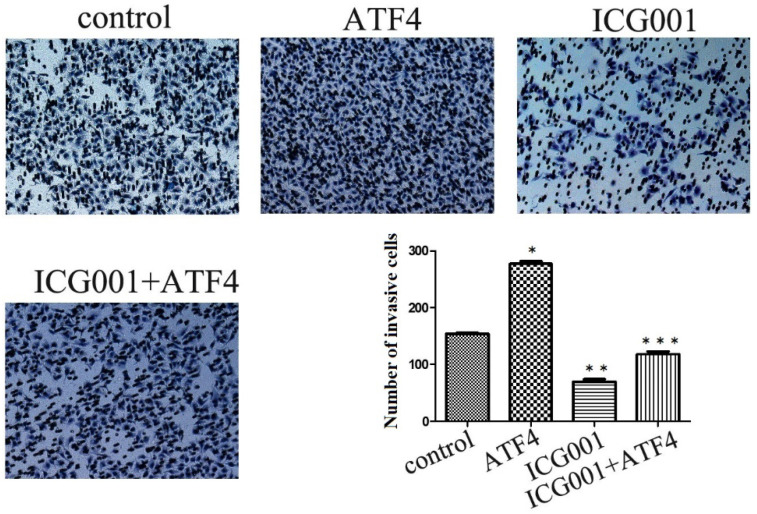
**The function of ATF4 to regulate cancer cell invasion was not dependent on Wnt/β-catenin signaling.** The matrigel invasion assay showed that ATF4 significantly promoted cancer cell invasion (*,* p*<0.05), while the Wnt/β-catenin signaling inhibitor ICG001 inhibited cancer cell invasion (**, *p*<0.05). Co-treatment with ICG001 and ATF4, however, significantly inhibited cancer cell invasion (*p* < 0.05). Compared to treatment with ICG001 alone, the invasion ability of cancer cells was significantly increased by treatment with ICG001 and ATF4 cDNA (*p* < 0.05) .The experiments were repeated at least 3 times.

The Wnt/β-catenin inhibitor ICG001 was employed to evaluate the molecular effects of ATF4. ICG001 mainly affects the β-catenin/CBP interaction and blocks the activation of the canonical Wnt pathway. Matrigel invasion assay shows that ICG001 significantly inhibited cancer cell invasion, whereas ATF4 significantly promoted cancer cell invasion (*p* < 0.05) (Figure 4). Co-treatment with ICG001 and ATF4, however, significantly inhibited cancer cell invasion (*p* < 0.05) (Figure 4). Compared to treatment with ICG001 alone, the invasion ability of cancer cells was significantly increased by treatment with ICG001 and ATF4 cDNA (*p* < 0.05) (Figure 4), indicating that the invasion-promoting function of ATF4 is not mediated only by Wnt/β-catenin signaling.

### ATF4-mediated modulation of Wnt/β-catenin signaling is abolished by ICG001

**Figure 5:**
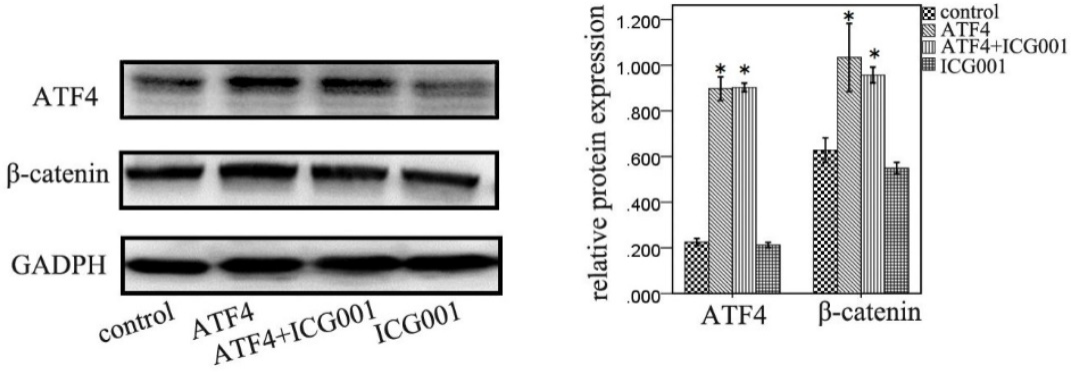
**The function of ATF4 and ICG001 to regulate β-catenin expression.** Both ATF4 and co-addition of ATF4 cDNA and ICG001 significantly upregulated β-catenin expression (*, *p*<0.05), while treatment with ICG001 only didn't significantly change the expression of β-catenin (*p*>0.05). The experiments were repeated at least 3 times.

**Figure 6:**
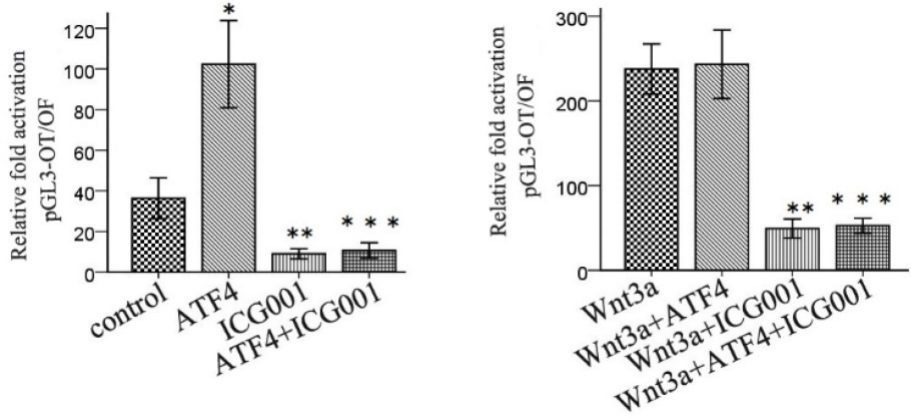
**ICG001 abolished the function of ATF4 to regulate the activity of Wnt/β-catenin signaling.** ATF4 significantly increased the activity of Wnt/β-catenin pathway (*, *p*<0.05), while when treatment with Wnt3a, ATF4 didn't further enhance the activity (*p*>0.05). ICG001 significantly inhibited the activity with or without Wnt3a (**, *p*<0.05). ICG001 nearly abolished the function of ATF4 to regulate the activity, with or without Wnt3a (***). The experiments were repeated at least 3 times.

ICG001 was used to investigate the function of ATF4 in regulating Wnt/β-catenin signaling. ATF4 significantly upregulated β-catenin expression (*p* < 0.05), whereas ICG001 did not significantly change the expression of β-catenin (*p* > 0.05) (Figure 5). Co-treatment of cells with ATF4 cDNA and ICG001 also significantly upregulated β-catenin expression (*p* < 0.05) (Figure 5). Dual-luciferase assays show that both ATF4 and Wnt3a significantly increased the activity of the canonical Wnt pathway (p < 0.05), whereas ICG001 significantly inhibited its activity with or without Wnt3a (*p* < 0.05) (Figure 6). ICG001 also abolished the function of ATF4 to promote canonical Wnt signaling activation with or without Wnt3a (Figure 6). Compared with treatment with Wnt3a alone, signaling activity did not change significantly in cells treated with both Wnt3a and ATF4 (Figure 6), which indicates that the function of ATF4 may be similar to that of Wnt3a in regulating the stability of the β-catenin protein.

## Discussion

ATF4 belongs to the CREB family 12. It has been shown to be induced by ER stress, oxidative stress, and hypoxia 8, 20. Some studies have shown that ATF4 plays an important role in tissue development 21, 22. Wang et al. showed that ATF4 in the nucleus regulates chondrocyte proliferation and differentiation 22. In addition, ATF4 was found to be involved in the regulation of metabolism 23. Here, we investigated ATF4 expression in human bronchial epithelial HBE cells and lung cancer cells. Our data show that ATF4 expression was mainly detected in nucleus in both HBE and cancer cells. In addition, ATF4 expression was upregulated in cancer cells compared to HBE cells in both the nucleus and cytoplasm. However, further studies indicate that there may be different molecular functions of ATF4 in HBE and cancer cells. Our previous study showed that ATF4 is upregulated in NSCLC and may contribute to cancer development. In addition, ATF4 is involved in regulating the biological behavior of tumor cells 24. In this study, we focused on the molecular links of ATF4 in NSCLC. Lung cancer is one of the most common kinds of cancer and also a leading type of death in cancer 1. To fully understand the molecular mechanism involved in its progression, it is critical to develop clinical targeted therapies.

ATF4 also has various functions in cancer cells 11, 25, although the issue remains controversial. Some studies have indicated the promotion of cancer progression by ATF4 12, 13, 15-17. In contrast, some studies have shown that ATF4 inhibits cancer progression 9, 14. Participation in the regulation of autophagy by ATF4 was reported 26, and ATF4 also plays a role in sensitivity to chemotherapy 9, 10. In the current study, our data indicate that ATF4 had a positive role in promoting cancer growth and invasion. When ATF4 was overexpressed, colony formation increased and invasive cancer cells were more numerous, indicating the important role of ATF4 in regulating lung cancer cell growth and invasion. ATF4 interacts with many molecules and signaling pathways 11-13, 15-17. Our study showed that ATF4 can upregulate β-catenin expression and the activity of Wnt/β-catenin signaling. To further investigate the underlying molecular mechanism, we used Wnt3a, a ligand that mainly activates canonical Wnt signaling, and the inhibitor ICG001 27. The data show that, when cancer cells were treated with Wnt3a, ATF4 did not further enhance the activity of canonical Wnt signaling, which suggests that ATF4 regulates Wnt/β-catenin signaling mainly through modulating β-catenin stability, similar to Wnt3a. The ICG001 inhibitor mainly affects Wnt/β-catenin signaling by preventing β-catenin/CBP interaction. In this study, we showed that the function of ATF4 to regulate the Wnt/β-catenin pathway was abolished by ICG001, which indicates that ATF4 mainly functions upstream of the β-catenin/CBP interaction. Yu's study showed that ATF4 promotes Wnt/β-catenin signaling by inhibiting the degradation of β-catenin 11, which is consistent with our study. Abnormal activation of Wnt/β-catenin signaling plays an important role in many cancers, including NSCLC. Our data indicate that ATF4 may regulate cancer cell growth and invasion at least partly by regulating Wnt/β-catenin signaling. MMP7 and cyclin D1 are two critical downstream molecules of Wnt/β-catenin signaling, which were also upregulated by ATF4 in this study. MMP7 belongs to the family of matrix metalloproteinases, which play important roles in cancer cell invasion by degrading various protein components of the extracellular matrix, thus destroying the normal histological barrier 28. Cyclin D1 is one of the cyclins, whose main function is to promote cell proliferation 29. Further studies to investigate the interaction between ATF4 and Wnt/β-catenin signaling may form the basis for the development of targeted therapies. Gao's study indicated that PSAT1 may be the main link between ATF4 and β-catenin 17. However, whether PSAT1 contributes to the molecular regulation of Wnt/β-catenin signaling by ATF4 in lung cancer cells is not clear. Moreover, our data also showed that the function of ATF4 to regulate cancer cell invasion was not dependent on Wnt/β-catenin signaling, and there may be other molecular mechanisms involved. Tameire's study indicated that ATF4 plays an important role in MYC driven tumor progression 16. The function of ATF4 in regulating cancer progression was also found to be related to PERK and CCL2 10,15. Whether these molecular links are involved in the function of ATF4 in lung cancer cells needs to be further investigated. In a summary, in this study we found that ATF4 is mainly located in the nucleus of lung cancer cells including A549, LK2 and H1299. ATF4 may promote the growth and invasion of lung cancer cells partly by promoting the activation of canonical Wnt pathway. These results will provide experimental basis for potential clinical targeted therapy.

## Abbreviations

ATF4: activating transcriptional factor 4
CREB: cAMP response element binding
NSCLC: non-small cell lung cancer
CBP: cyclic adenosine monophosphate response element-binding protein
SCC: squamous cell carcinoma
